# Supplementary material for: Myosin-dependent short actin filaments contribute to peripheral widening in developing stereocilia
Source: Res Sq. 2024 Dec 5:rs.3.rs-5448262. Preprint. [Version 1] doi: 10.21203/rs.3.rs-5448262/v1 (PMC11643313; doi:10.21203/rs.3.rs-5448262/v1)
Supplement: 1 [file NIHPPRS5448262V1-supplement-1.pdf]

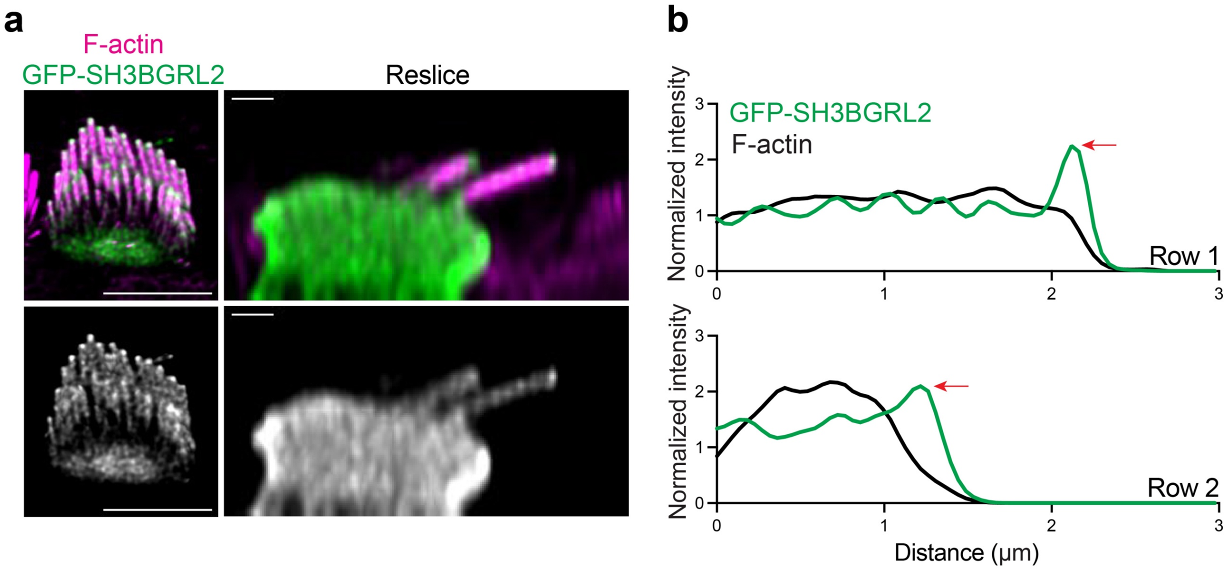

Supplementary Fig. 1: Localization of transfected EGFP-SH3BGRL2 in IHCs.
a, Representative images of EGFP-SH3BGRL2 distribution in transfected P5 IHCs. Left panels are 2D projections of x-y slices (scale bar represents 5 μm). Right panels are projections of x-z reslices to show the side view of stereocilia (scale bar represents 1 μm). **b,** The fluorescence distribution of EGFP-SH3BGRL2 and phalloidin stained F-actin measured on the line scan of a stereocilium in (**a**)**.** Red arrows indicate that the intensity of EGFP-SH3BGRL2 reaches near its peak at stereocilia tips.

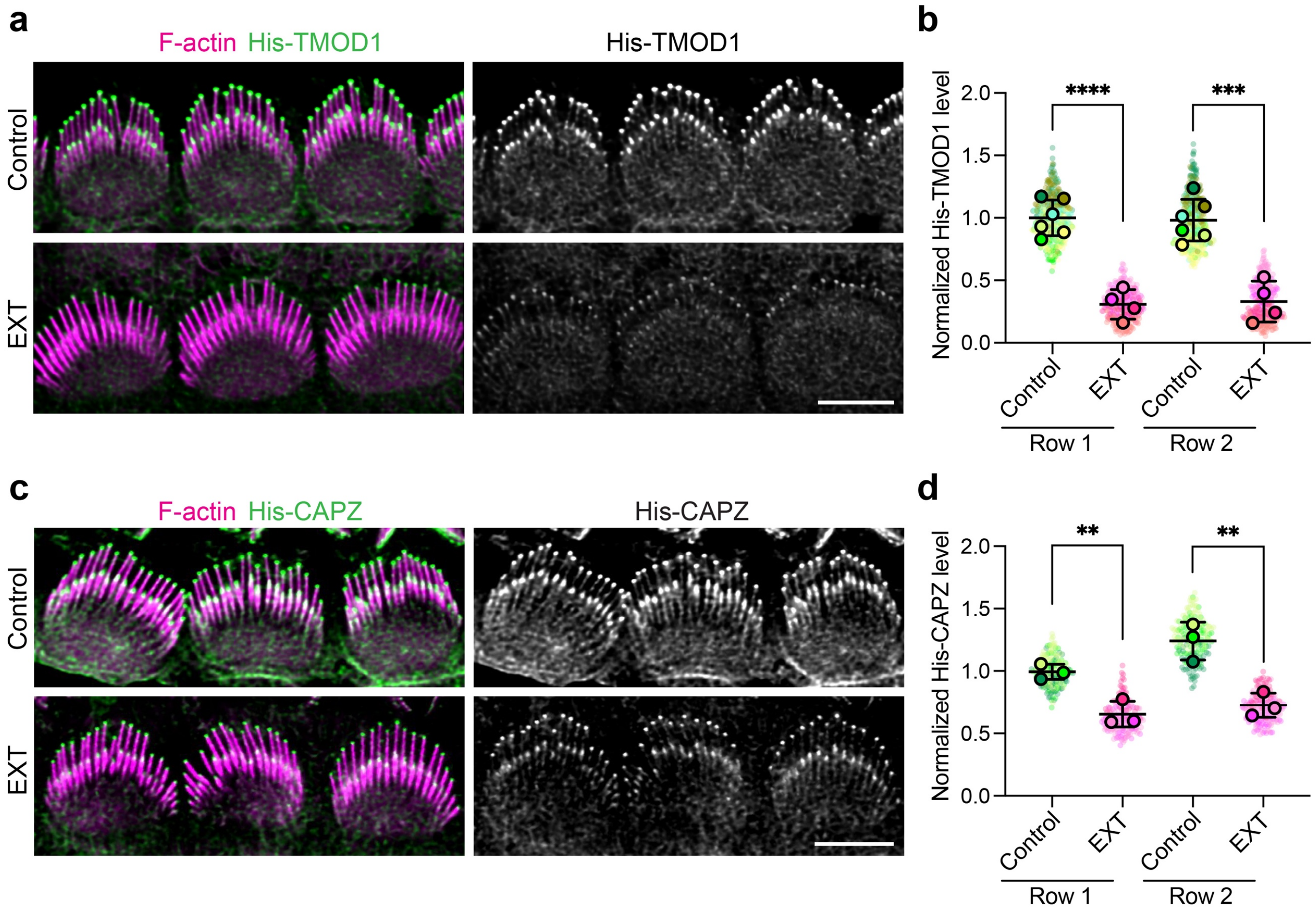

Supplementary Fig. 2: Tip filaments are separate from the stereocilia core filaments.

a, His-TMOD1 staining (green, grey) in P5 IHCs after high salt extraction (EXT); F-actin was stained with phalloidin (magenta). **b**, Quantification of His-TMOD1 level from row 1 and row 2 stereocilia tips. The fluorescence intensity was normalized to the average fluorescence intensity of row 1 control treatment. **c,** His-CAPZ staining (green, grey) in P5 IHCs after high salt extraction; F-actin was stained with phalloidin (magenta). **d,** Quantification of His-CAPZ level from row 1 and row 2 stereocilia tips. Smaller circles represent stereocilia; larger open circles represent cochleae (N). *P* values for two-tailed unpaired *t* tests comparing N are indicated (**, $P < 0.01$; ***, $P < 0.001$; ****, $P < 0.0001$). Results from 3-6 mice were averaged and plotted ± SD. All scale bars represent 5 μm.

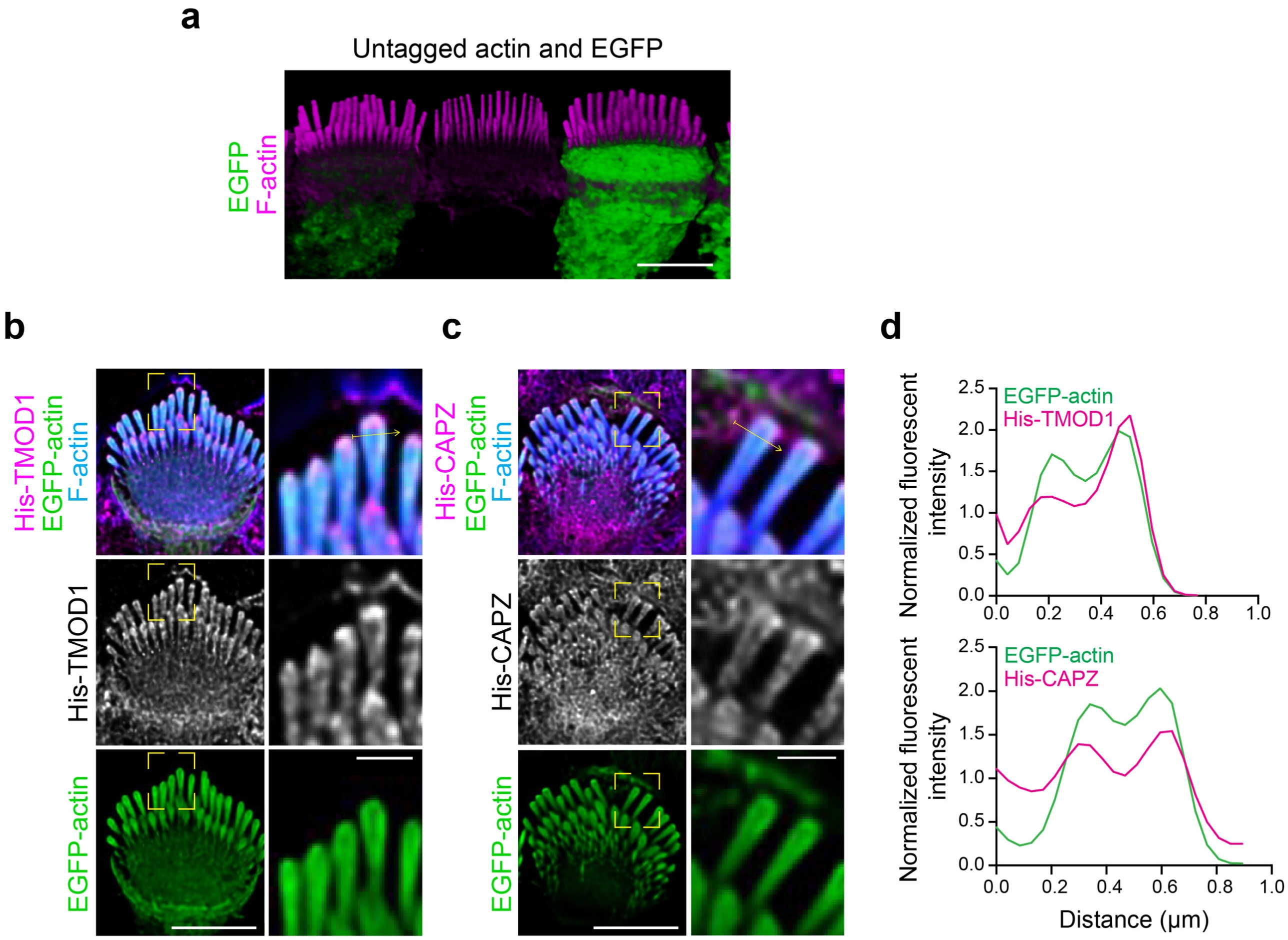

Supplementary Fig. 3: Untagged actin overexpression promotes stereocilia widening.
a, Representative image showing a 3D reconstruction of P5 IHCs transfected by Actin-IRES-EGFP. Transfected IHCs, identified by cytoplasmic EGFP, exhibit wider stereocilia compared to a neighboring untransfected IHC. F-actin is stained by phalloidin (magenta). **b-c,** Representative images showing His-TMOD1 (**b**) or His-CAPZ staining (**c**) (magenta, grey) in widened stereocilia (blue) from IHCs transfected with EGFP-actin (green) (scale bar represents 5 μm). Regions marked by yellow boxes are magnified to the right panels (scale bar represents 1 μm). The yellow arrows indicate line scans graphed in (**d**). **d,** The fluorescence distribution of His-TMOD1 or His-CAPZ (magenta) with EGFP-actin (green). The fluorescence intensity was normalized to the average fluorescence intensity of each label.

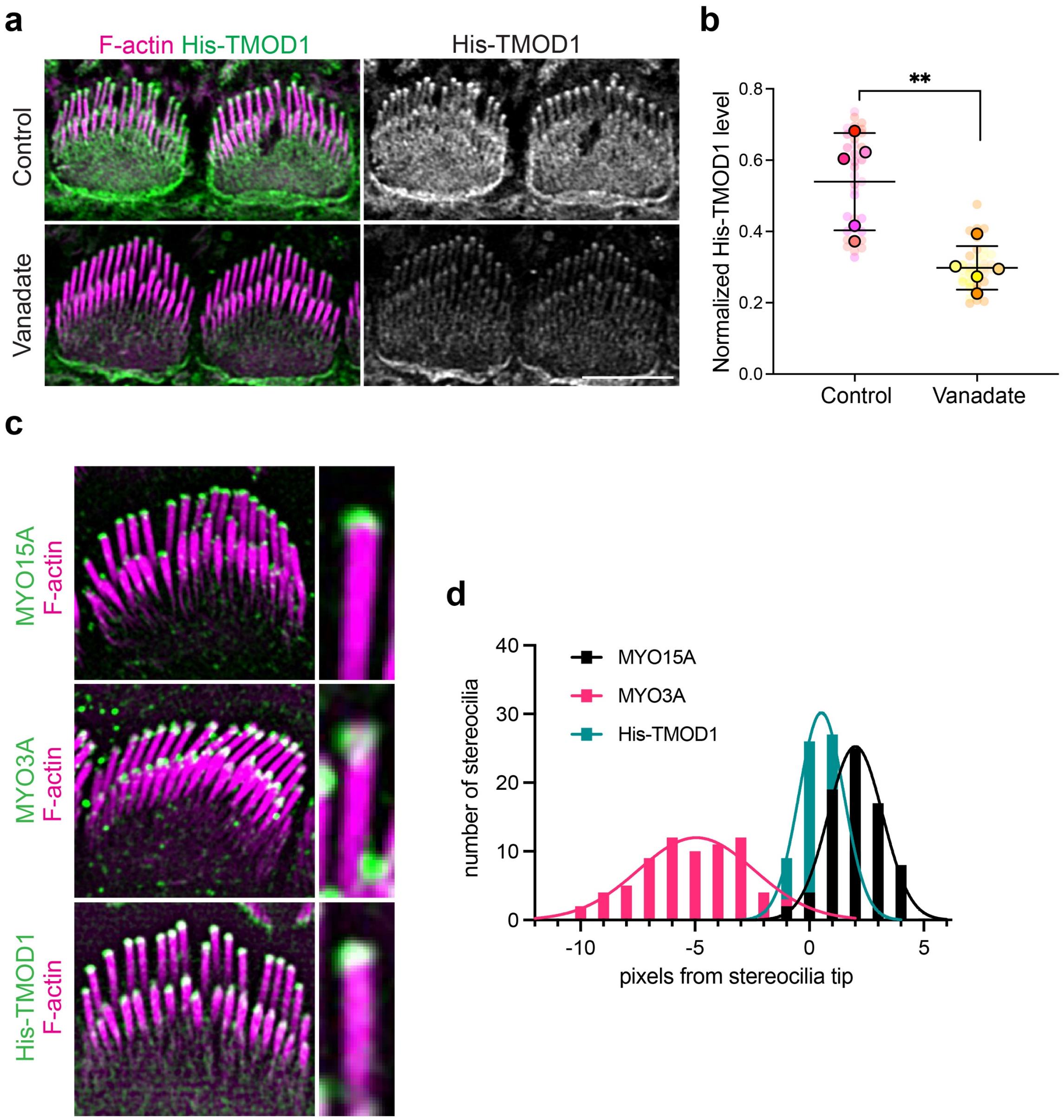

Supplementary Fig. 4: Tip filaments are likely stabilized or produced by myosins.

a, His-TMOD1 staining (green, grey) in P5 IHCs incubated with or without vanadate. **b,** Quantification of His-TMOD1 level at stereocilia tips in vanadate-treated and untreated IHCs. Smaller circles represent stereocilia; larger open circles represent cochleae (N). *P* values for two-tailed unpaired *t* tests are indicated based on N (**, $P < 0.01$). Results from 5 cochleae were averaged and plotted ± SD. **c,** Representative lattice SIM images showing the localization of endogenous MYO15A, MYO3A, and His-TMOD1 (green) in IHC bundles. The magnified insets show the localization of each protein at row 1 stereocilia tips compared to phalloidin-stained F-actin (magenta). **d,** A frequency histogram showing the pixel offset of MYO15A (black), MYO3A (red) and His-TMOD1 (blue) from the actin core. The histogram of each probe is fitted in a Gaussian curve. Mean offsets for peak of the Gaussian curves: MYO15A, 62 nm; MYO3A, -153 nm; His-TMOD1, 16 nm. R-squared value of the fit: MYO15A, 0.985; MYO3A, 0.921; His-TMOD1, 0.996. The stereocilia tip was defined as being the point where phalloidin intensity reached the average value in the tip region.

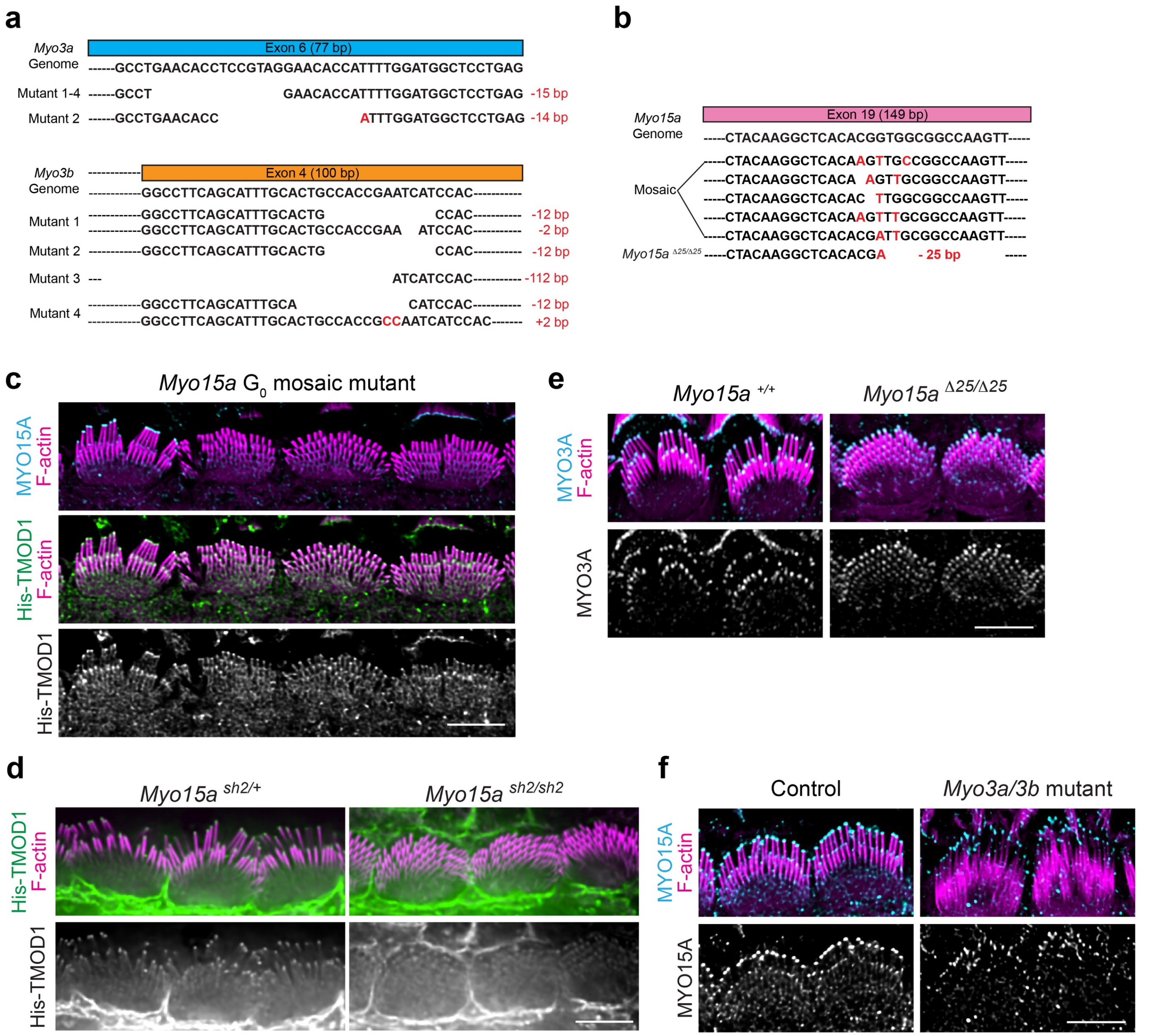

Supplementary Fig. 5: Characterization of mutant *Myo3* and *Myo15a* alleles.

a-b, Schematics showing mutant alleles detected by nanopore sequencing of genomic DNA from pups that were mutated by CRISPR/Cas9 gRNAs targeting *Myo3a* and *Myo3b* concurrently (**a**) or *Myo15a* (**b**) that were delivered to mouse embryos by the iGONAD method. **c,** Mutant *Myo15a* exhibiting a mosaic phenotype. MYO15A (cyan) and His-TMOD (green, grey) at highest at the tips of the hair cell at left that retains normal stereocilia lengths, but His-TMOD1 is reduced at the tips of stereocilia on neighboring hair cells that are short and lack MYO15A. **d,** His-TMOD1 staining (green, grey) in P4 IHCs from mice heterozygous or homozygous for the *sh2* loss-of-function mutation. Homozygous mutants have short stereocilia with reduced His-TMOD1 staining. **e,** MYO3A immunostaining (cyan, grey) of either wildtype or *Myo15a*$^{\Delta25/\Delta25}$ P4 IHC stereocilia. **f,** MYO15A immunostaining (cyan, grey) of either wildtype or *Myo3a;Myo3b* mutant P4 IHC stereocilia. F-actin was stained with phalloidin (magenta) in (**c-f**).

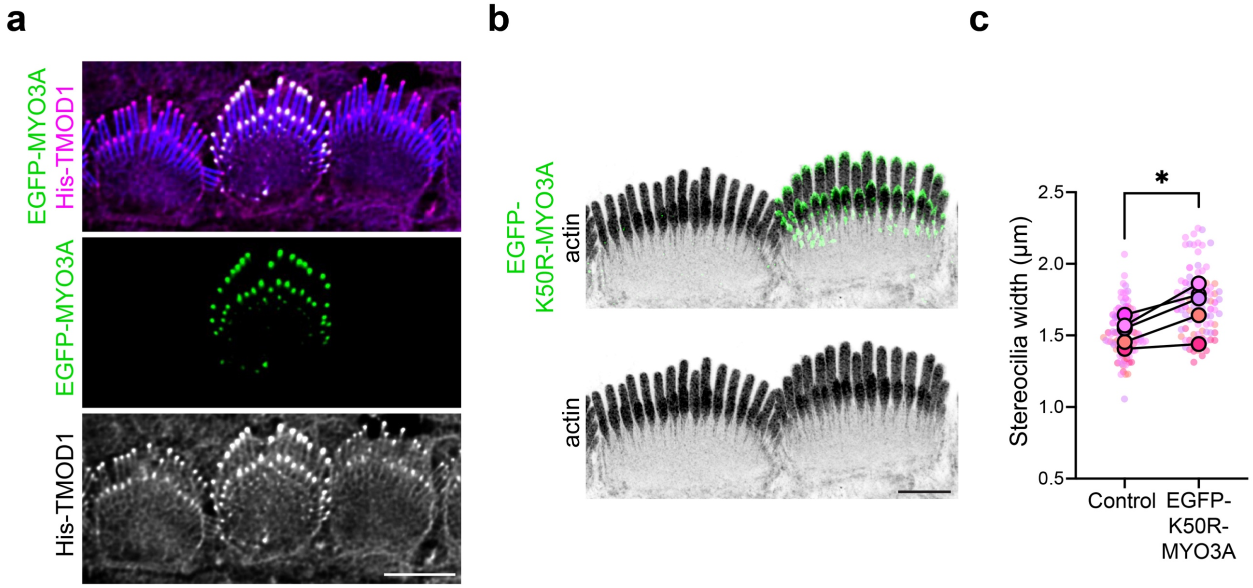

Supplementary Fig. 6: Overexpression of EGFP-MYO3A and stereocilia widening.
a, Representative images comparing His-TMOD1 staining (magenta, grey) in P5 IHCs transfected with wildtype (WT) EGFP-MYO3A (green) compared to neighboring untransfected cells. F-actin is stained with phalloidin (blue). **b,** Representative expansion microscopy images of an EGFP-K50R-MYO3A transfected IHC adjacent to an untransfected IHC at P5. EGFP-K50R-MYO3A (green) was stained with an antibody to EGFP, and actin (grey) was stained with anti-γ-actin antibody. **c**, Quantification of stereocilia width in EGFP-MYO3A-K50R transfected cells and untransfected cells. Smaller represent individual stereocilia and larger open circles represent cochleae. Results were collected from 5 cochleae. *P* values for two-tailed paired *t* tests are indicated (*, $P < 0.05$), comparing averages of cochleae. Scale bars in all panels represents 5 µm.
